# Supplementary material for: Chemical inhibition of SUMOylation activates the FSHD locus
Source: Sci Rep. 2026 Jan 9;16:3572. doi: 10.1038/s41598-025-33624-0 (PMC12847776; doi:10.1038/s41598-025-33624-0)
Supplement: Supplementary file 1 — Supplementary Information 1. [file 41598_2025_33624_MOESM1_ESM.docx]

**Supplementary information**

**Materials & Methods**

**Cell differentiation and pharmacological treatment**

For human immortalized myoblasts, once 90% confluency was reached, Growth Medium was replaced for 6 days by Differentiation Media (DM) composed of 4:1 DMEM/Medium 199 supplemented with 2% Horse Serum (Gibco) to induce final differentiation. Cells were treated with DMSO or TAK-981 (HY-111789, MedChemExpress) diluted in Differentiation Medium from day 4 to day 6 and changed every day.

Differentiation of hiPSC clones was performed as described ^1,2^. Briefly, at day 0 they were switched for a differentiation medium (neurobasal medium supplemented with N2 1X, B27 1X, penicillin/streptomycin 1X, non-essential amino-acid 1X, Glutamax 1X ; Life Technologies) changed every day, containing ITS-A 1X (51300044, Life Technologies), LDN193189 0,5µM (SML0559, Sigma-Merck) and CHIR99021 3µM (5ML-1046, Sigma-Merck). From day 6 to day 8, medium is replaced by a differentiation medium containing LDN193189, IGF1 4ng/mL, HGF 10ng/mL (100-39, Peprotech) and β-mercaptoethanol 90,8µM (31350010, ThermoFisher). At day 8, medium is changed for a differentiation medium supplemented only with IGF1and β-mercaptoethanol for four days. At day 12, a differentiation medium was used supplemented with IGF1, β-mercaptoethanol and DAPT 10µM (Tocris Biosciences) until day 17. From day 17 to the end of experiment at day 30, medium was changed every day for differentiation medium supplemented with IGF1. Cells were treated from day 15 to day 30 with DMSO or TAK-981 diluted in the appropriate differentiation medium and changed every day.

**Quantitative real-time PCR**

Total RNA was collected at the end of differentiation process at day 6 for human immortalized myoblasts and day 30 for hiPSC clones. Total RNA was extracted using TriZol reagent (Invitrogen) following manufacturer’s instructions and quantiﬁed using a spectrophotometer (ND-1000, Nanodrop Technologies). 2µg of RNA were used to synthetize cDNA using SuperScript IV kit (18091050, Invitrogen) preceded by a step of genomic DNase digestion with ezDNase (11766051, Invitrogen). Quantitative PCR was performed on a CFX96 real Time System Thermal Cycler (Bio-Rad) using SYBR Green PCR Mastermix (Applied Biosystems) and specific primers available in Table S1.

**Immunoblots**

Protein extracts were collected at the end of differentiation process at day 6 for human immortalized myoblasts and day 30 for hiPSC clones immediately in Laemmli buffer (Bio-Rad) 1X with β-mercaptoethanol to avoid rapid deSUMOylation of proteins. After boiling and sonication steps, protein dosage was performed using Ionic Detergent Compatibility Reagent for Pierce 660nm Assay (22660, Pierce) and a SkanIt (ThermoFisher) spectrophotometer at 660nm. Equal quantities of protein extracts were loaded and were verified after transfer (Bio-Rad) by a Ponceau staining on nitrocellulose membranes. For SUMO immunoblots, Bis-Tris polyacrylamide 4-12% gels (Bio-Rad) were used. For endogenous SMCHD1 immunoblots, homemade gels at 6.5% Bis-acrylamide were prepared.

Anti-SUMO1 (ab32058, Abcam; 1/1000), anti-SUMO2/3 8A2 (ab81371 Abcam; 1/1000), anti-Vinculin (ab18058; Abcam; 1/1000), anti-SMCHD1 (ab175235; Abcam; 1/666) were applied as primary antibodies, followed by the secondary antibody at a 1:10,000 dilution, IRDye 800CW goat anti-rabbit (926-32211, LI-COR) and IRDye 680LT goat-anti-mouse (926-68020, LI-COR). Immunoreactivity was detected with an OdysseyTM Infrared-Imaging System (LI-COR), according to the OdysseyTM Western Blotting Protocol.

***DUX4* electrophoresis**

qPCR products resulted from *DUX4* RT-qPCR amplification were loaded on 2% agarose gels (G800802; Invitrogen) and imaged using Gel Doc XR System (Bio-Rad) device.

**Sodium bisulfite sequencing**

For bisulfite modification, 1 μg of genomic DNA was denatured for 30 minutes at 37°C in NaOH 0.4N and incubated overnight in a solution of 3M Sodium bisulfite pH5 and 10mM Hydroquinone using previously described protocol ^3^. Converted DNA was then purified using the Wizard DNA CleanUp kit (Promega) following manufacturer’s recommendation and precipitated by ethanol precipitation for 5 hours at -20°C. After centrifugation, DNA pellet was resuspended in 40μL of water and stored at -20°C until use. Converted DNA was amplified using the DR1 primers avoiding the presence of CpGs in the primer sequence in order to amplify both methylated and unmethylated DNA with the same efficiency^4^. After sequencing of the PCR fragments, the methylation level was calculated as described^5^ and corresponds to the global level of methylation for each biological sample in a given region calculated as the ratio of methylated CpG with the number of aligned CpG for all sequences and CpG positions for a given biological sample.

**Immunofluorescence Assay**

Immunofluorescence assays were performed as follows: cells were grown on coverslips and fixed for 20 min with 4% paraformaldehyde diluted in 1x PBS. After washing with PBS (3 × 5 min), the cells were incubated for 5 min at room temperature in a permeabilization solution (PBS with 3% BSA and 0.5% Triton X-100). They were then incubated for 1 to 2 hours at room temperature in a blocking solution (PBS with 3% BSA and 0.1% Triton X-100). Cells were subsequently incubated overnight at 4°C in blocking solution containing the primary antibodies: Myogenin (1:200; Abcam, Cat. No. ab1835) and Titin (1:200; Thermofisher, Cat. No. 27867-1-AP). After three washes with PBS containing 0.2% Tween-20 (3 × 5 min), the slides were incubated for 2 hours at room temperature with Alexa Fluor 488 and 546 secondary antibodies (1:1000; Thermofisher Cat. No A11003 (546); Cat. No A11008 (488)) diluted in blocking solution. DAPI staining was performed during the first wash in PBS with 0.2% Tween-20 for 5 min, followed by two additional washes in PBS-T (2 × 5 min). The slides were mounted using Fluoromount-G (Thermofisher, Cat. No. 00-4958-02). Images were acquired using a Zeiss Axio Observer Z.1 microscope system. Cell counting was performed manually using the Zen Lite software. Each image was examined individually, and cells were counted based on the specific fluorescence signals corresponding to the different markers selected. To ensure the accuracy of the results, multiple fields were evaluated per sample, with a minimum of five fields captured for each condition.

**Quantification and statistical analysis**

For RT-qPCR analysis, fold-change values of gene expression were obtained using the 2^-ΔΔCt^ method. Briefly, ΔCt values were first calculated relative to the average Ct of three housekeeping genes (*PPIA, HPRT, GUSB*), then normalized to the untreated condition (0).

Statistical analyses of RT-qPCR data were performed using ordinary one-way ANOVA on the relative ΔCt values. When significancy is observed, multiple comparisons were conducted with a Dunnett T3 test.

For nuclei counts in myotubes, statistical analyses were executed using a non-parametric Kruskall-Wallis test. When significancy is observed, multiple comparisons were conducted with a Dunn’s test.

All statistical analyses were realized with GraphPad/Prism 10, with validation of the bioinformatics and biostatistics hub of Institut Pasteur.

**Legends of Supplementary Figures**

**Supplementary Figure 1: Validation of TAK-981 induced hypoSUMOylation in hTERT immortalized myotubes**

(**a**) Immunoblots for SUMO1 and SUMO2/3 in control (AB1079) myotubes. Actin is used as a loading control.

(**b**) Immunoblots for SUMO1 and SUMO2/3 in FSHD1-patient derived myotubes (AB1080). Actin is used as a loading control.

(**c**) Electrophoresis of *DUX4* qPCR products in all tested control (12UBCT, AB1079) and FSHD1-patient derived myotubes (12ABCT, AB1080) treated with indicated concentrations of TAK-981 from day 4 to day 6 of differentiation.

**Supplementary Figure 2: Validation of TAK-981 induced hypoSUMOylation in hiPSC-derived muscle cells.**

(**a**-**d**) Immunoblots for SUMO1 and SUMO2/3 in control AG08498 (a), control 17706-L (b), FSHD1 17796-S (c) and FSHD2 14586 (d) hiPSC-derived myofibers. Actin is used as a loading control.

**Supplementary Table S1. List of primers used for RT-qPCR analysis**

| **Gene** | **Sequence Forward** | **Sequence Reverse** |
| --- | --- | --- |
| *HPRT* | TGATAGATCCATTCCTATGACTGTA | CAAGACATTCTTTCCAGTTAAAGTTG |
| *PPIA* | ATGCTGGACCCAACACAAAT | TCTTTCACTTTGCCAAACACC |
| *GUSB* | CCGAGTGAAGATCCCCTTTTTA | CTCATTTGGAATTTTGCCGATT |
| *DUX4* | AGGCGCAACCTCTCCTAGAAA | GCTCCTCCAGCAGAGCCCGGTATTC |
| *DUX4c* | CACCAGAGTTTCAGCAAAAG | GTGTTCTTCCTGGCTGAG |
| *ZSCAN4* | TGGAAATCAAGTGGCAAAAA | CTGCATGTGGACGTGGAC |
| *TRIM43* | ACCCATCACTGGACTGGTGT | CACATCCTCAAAGAGCCTGA |
| *MBD3L2* | GCGTTCACCTCTTTTCCAAG | GCCATGTGGATTTCTCGTTT |
| *LEUTX* | CTTCAAAGCTACAACTTGATCTATCC | AGTCTCCTCCTTCTTCACTGA |
| *FRG2* | CGCACCTTTCACTTGAGCTT | GAATGGGAGAAGGCGGTCT |
| *FRG1* | TTGTTGGAATCTGGTGGACA | CCATTGTCGAGTGCATGTATATAGG |
| *FAT1* | CATTAGAGATGGCTCTGGCG | ATGGGAGGTCGATTCACG |
| *WWC2* | TGACAATATGGCAGTTCGCCCCA | TCACTGTCACTCCGATTTAACCTGC |
| *MYOD* | CTCCGACGGCATGATGG | TGCTGGACAGGCAGTCTA |
| *MYOG* | CAGCGAATGCAGCTCTCA | GGTTGTGGGCATCTGTAGG |
| *MYH2* | TTCTCAGGCTTCAAGATTTGG | CTGGAGCTTGCGGAATTTAG |
| *MYH3* | ATCGTGAAAACCAGTCCATTCT | TTGGCCAGGTCCCCAGTAGCT |

­

**References**

1 Delourme, M., Broucqsault, N., Mazaleyrat, K. & Magdinier, F. Production of Innervated Skeletal Muscle Fibers Using Human Induced Pluripotent Stem Cells. *Methods Mol Biol*, doi:10.1007/7651_2020_334 (2020).

2 Mazaleyrat, K. *et al.* Multilineage Differentiation for Formation of Innervated Skeletal Muscle Fibers from Healthy and Diseased Human Pluripotent Stem Cells. *Cells* **9**, doi:10.3390/cells9061531 (2020).

3 Magdinier, F. *et al.* Regional methylation of the 5' end CpG island of BRCA1 is associated with reduced gene expression in human somatic cells. *Faseb J* **14**, 1585-1594 (2000).

4 Hartweck, L. M. *et al.* A focal domain of extreme demethylation within D4Z4 in FSHD2. *Neurology*, doi:10.1212/WNL.0b013e31827f075c (2013).

5 Roche, S. *et al.* Methylation hotspots evidenced by deep sequencing in patients with facioscapulohumeral dystrophy and mosaicism. *Neurol Genet* **5**, e372, doi:10.1212/NXG.0000000000000372 (2019).
