# Supplementary figures and images for: Chemical inhibition of SUMOylation activates the FSHD locus
Source: Sci Rep. 2026 Jan 9;16:3572. doi: 10.1038/s41598-025-33624-0 (PMC12847776; doi:10.1038/s41598-025-33624-0)

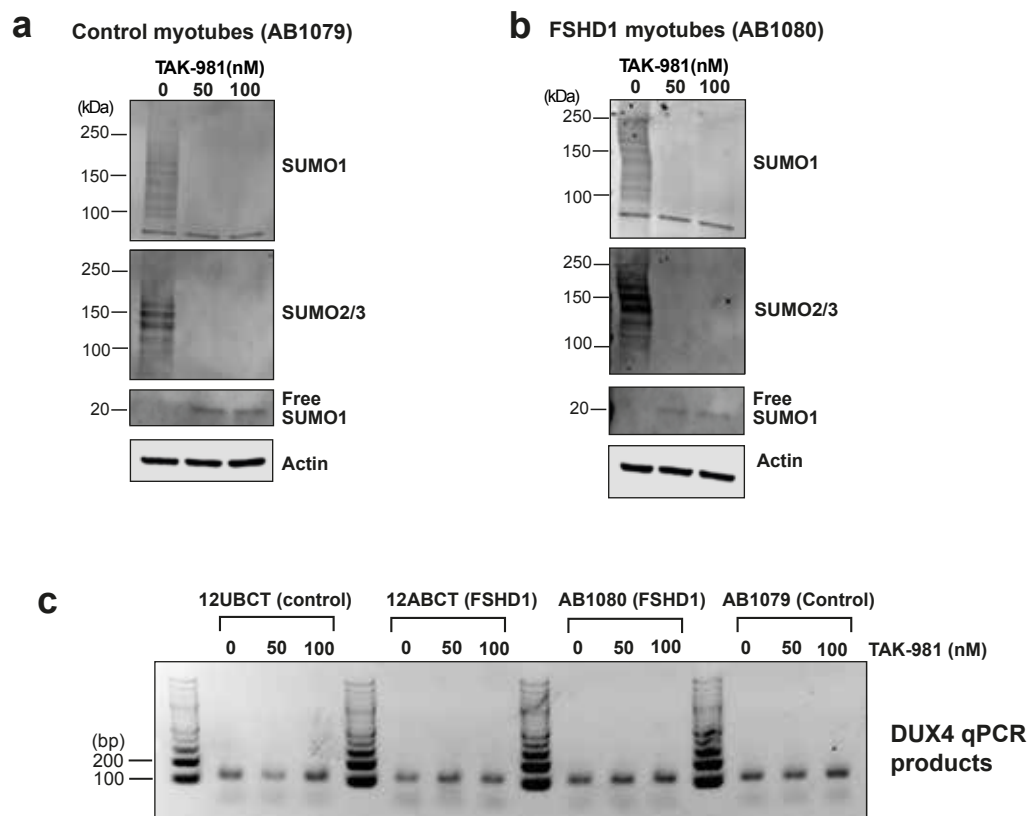

Supplementary figure 1

Supplement: Supplementary file 2 — Supplementary Information 2. [file 41598_2025_33624_MOESM2_ESM.pdf]

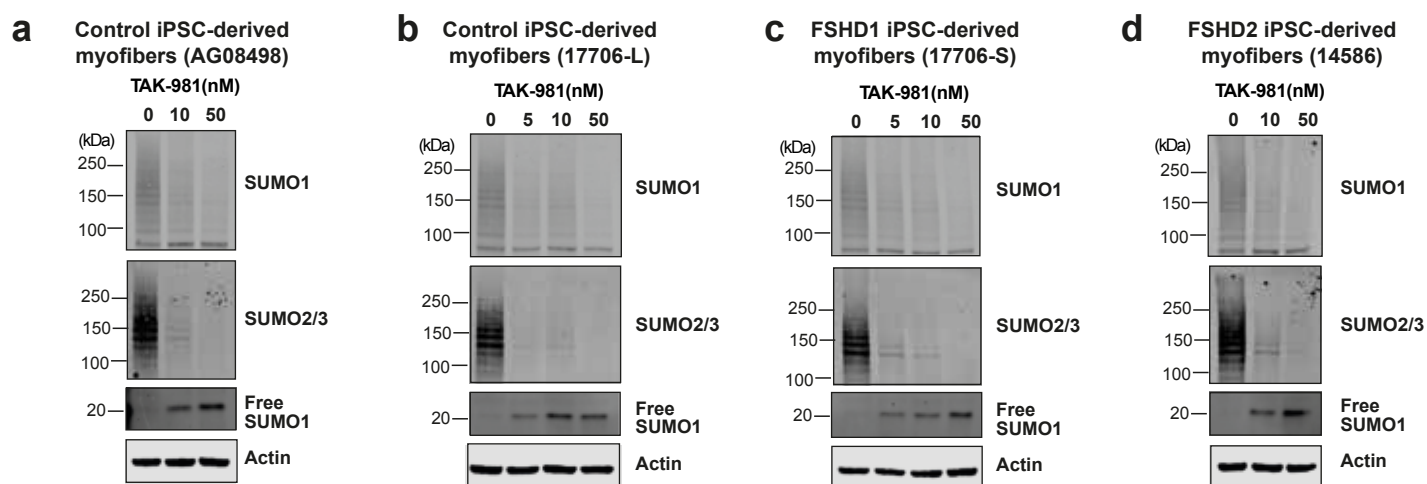

Supplementary figure 2

Supplement: Supplementary file 3 — Supplementary Information 3. [file 41598_2025_33624_MOESM3_ESM.pdf]
